# Mechanisms of ferroptosis

**DOI:** 10.1007/s00018-016-2194-1

**Published:** 2016-04-05

**Authors:** Jennifer Yinuo Cao, Scott J. Dixon

**Affiliations:** Department of Biology, Stanford University, 337 Campus Dr., Stanford, CA 94305 USA

**Keywords:** Cell death, Iron, Reactive oxygen species, Glutathione, Cancer, RAS, Glutathione peroxidase 4, Erastin, Sorafenib, Ferrostatin-1, Polyunsaturated fatty acid

## Abstract

Ferroptosis is a non-apoptotic form of cell death that can be triggered by small molecules or conditions that inhibit glutathione biosynthesis or the glutathione-dependent antioxidant enzyme glutathione peroxidase 4 (GPX4). This lethal process is defined by the iron-dependent accumulation of lipid reactive oxygen species and depletion of plasma membrane polyunsaturated fatty acids. Cancer cells with high level RAS-RAF-MEK pathway activity or p53 expression may be sensitized to this process. Conversely, a number of small molecule inhibitors of ferroptosis have been identified, including ferrostatin-1 and liproxstatin-1, which can block pathological cell death events in brain, kidney and other tissues. Recent work has identified a number of genes required for ferroptosis, including those involved in lipid and amino acid metabolism. Outstanding questions include the relationship between ferroptosis and other forms of cell death, and whether activation or inhibition of ferroptosis can be exploited to achieve desirable therapeutic ends.

## Introduction

**Fig. 1:**
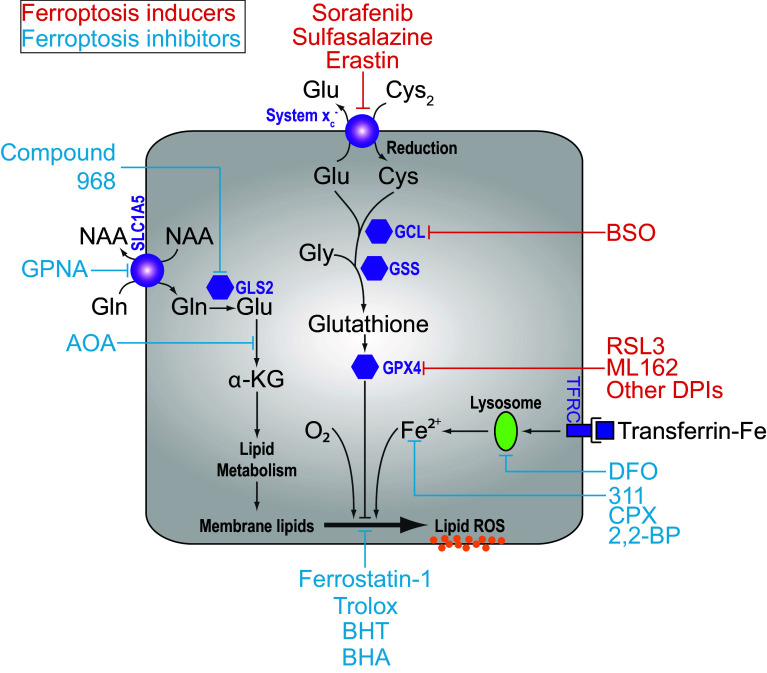
Overview of the ferroptosis pathway. In many cells, cystine (Cys_2_) import via system $x_{\text{c}}^{ - }$ is required for glutathione synthesis, and the function of glutathione peroxidase 4 (GPX4). GPX4 activity prevents the accumulation of lipid ROS that are lethal to the cell. Treatment blocks cystine uptake, ultimately depleting the cell of glutathione and inhibiting the function of GPX4. Direct inhibition of the rate-limiting glutathione synthetic enzyme glutamate-cysteine ligase (GCL) using buthionine-(*S*,*R*)-sulfoximine (BSO) can also lead to the same iron- and ROS-dependent ferroptotic phenotype. Other small molecule inducers of ferroptosis are indicated in * red*, while suppressors of ferroptosis are in * blue*. *GCL* Glutamate cysteine ligase, *GSS* glutathione synthetase, *Cys* cysteine, *Glu* glutamate, *Gly* glycine, *Gln* glutamine. *α-KG* alpha-ketoglutarate, *GPNA* l-g-glutamyl-*p*-nitroanilide, *AOA* amino oxyacetate, *BHA* butylated hydroxyanisole, *BHT* butylated hydroxytoluene, *DFO* deferoxamine, *2,2-BP* 2,2-bipyridyl, *CPX* ciclopirox

Regulated cell death (RCD) is essential for normal development and the maintenance of homeostasis. RCD can proceed through apoptosis or one of several non-apoptotic cell death pathways, including the recently described process of ferroptosis [1–5]. Ferroptosis is an oxidative, iron-dependent form of cell death that is distinct from apoptosis, classic necrosis, autophagy and other forms of cell death [5] (Table 1). Ferroptosis is triggered by inactivation of cellular glutathione (GSH)-dependent antioxidant defenses, leading to the accumulation of toxic lipid ROS (L-ROS) [5, 6] (Fig. 1). This process has recently been implicated in the pathological cell death of brain tissues exposed to high levels of glutamate (Glu) as well as kidney and heart tissues subjected to ischemia–reperfusion injury [5, 7–10]. In the context of cancer, ferroptosis may act as an endogenous tumor suppressive mechanism downstream of p53 [11]. It may also be possible to use small molecule activators of ferroptosis to selectively eliminate cancer cells with mutations in the RAS-RAF-MEK pathway, although this remains controversial [6, 12, 13]. It is, therefore, of great interest to understand how this novel RCD pathway is regulated.

**Table 1:** A comparison of features associated with various forms of apoptotic and non-apoptotic cell death

Cell death process	Death stimulus	Initiator	Mediator	Executioner	Hallmarks	Inhibitors
Apoptosis						
Extrinsic Pathway	• Death ligand binding to receptors of the tumor necrosis factor (TNF) superfamily (e.g. FasL/FasR, TNFα/TNFR1, Apo3L/DR3, Apo2L/DR4, Apo2L/DR5)	Activation of TNF death receptors Recruitment of cytoplasmic adaptor proteins (e.g. FADD and TRADD)	Formation of death-inducing signaling complex (DISC), consists of FADD and procaspase-8	Caspase 3 and endonuclease activation	Caspase activation Cytochrome c release Plasma membrane blebbing Nuclear fragmentation Chromatin condensation and margination Externalization of phosphatidylserine on the plasma membrane	Caspase inhibitors
Intrinsic Pathway	• DNA damage • Growth factor withdrawal • Hypoxia • Viral infection • Toxins • Hyperthermia	Loss of mitochondrial transmembrane potential Mitochondrial outer membrane permeabilization (MOMP) Release of pro-apoptotic proteins into cytosol (e.g. cytochrome c)	Formation of apoptosome, consist of cytochrome c, Apaf-1 and procaspases-9 Caspase 9 activation			
Necroptosis	• Death ligand (e.g. Fas, TNFα, TRAIL) binding to TNF receptor in caspase-inhibited cells	TNFR1 activation Recruitment of TRADD and RIPK1	In the absence of caspase 8, formation of the necrosome by phosphorylation of RIPK1 and RIPK3	Phosphorylation and oligomerization of MLKL proteins that insert into and permeabilize the plasma membrane	Plasma membrane permeabilization Swelling of organelles (e.g. mitochondria)	Necrostatins (e.g. Nec-1) Necrosulfonamide
Ferroptosis	• Inhibition of cystine import (e.g. erastin, SAS, glutamate) • Glutathione depletion (e.g. BSO) • GPX4 inactivation (e.g. RSL3) • AA depletion in presence of serum and glucose	System $x_{\text{c}}^{ - }$ inhibition Inhibition of GCL Inhibition of GPX4	Unknown (possibly not needed)	Unchecked lipid peroxidation and oxidative lipid fragmentation; normally opposed by GPX4	Lipid peroxidation Iron-dependence	Lipophilic antioxidants (e.g. Fer-1, vitamin E) Iron chelators (e.g. DFO, CPX)
Oxidative glutamate toxicity	• High concentrations of extracellular glutamate	Inhibition of system $x_{\text{c}}^{ - }$ result in glutathione depletion	Mitochondrial ROS production Opening of cyclic GMP-gated Ca^2+^ channels on plasma membrane	Ca^2+^-dependent activation of calpains triggering lysosomal membrane permeabilization, processing of BID and release of AIF1	Mitochondrial ROS production Ca^2+^ influx Oxidative stress	Antioxidants (e.g. vitamin E, idebenone) PD150606, a calpain inhibitor
Autophagic cell death	• In *Bax*/*Bak* double knockout MEFs or cells overexpressing antiapoptotic Bcl-2 or Bcl-x_L_ proteins, treatment with etoposide, staurosporine, thapsigargin	Upregulation of Atg5 and Atg6	Unknown	Autophagosome and autolysosome formation	Large-scale sequestration of cytoplasmic contents in autophagosome and autolysosomes	Autophagy inhibitors (e.g. 3-MA, wortmannin)
Parthanatos	• UV • Alkylating agents (e.g. MNNG) • Ca^2+^ influx • ROS	Hyperactivation of Poly(ADP-ribose) polymerase 1 (PARP1)	Release of apoptosis-inducing factor (AIF) into the cytoplasm	Unknown mechanism that activates endonuclease	NAD^+^ and ATP depletion	PARP1 inhibitors

*GSH* reduced glutathione, *GCL* glutamate-cysteine ligase, *GPX4* glutathione peroxidase 4, *AA* amino acid, *FADD* Fas-associated protein with death domain, *TRADD* tumor necrosis factor receptor type 1-associated DEATH domain protein

## The recognition of ferroptosis as a unique form of RCD

**Fig. 2:**
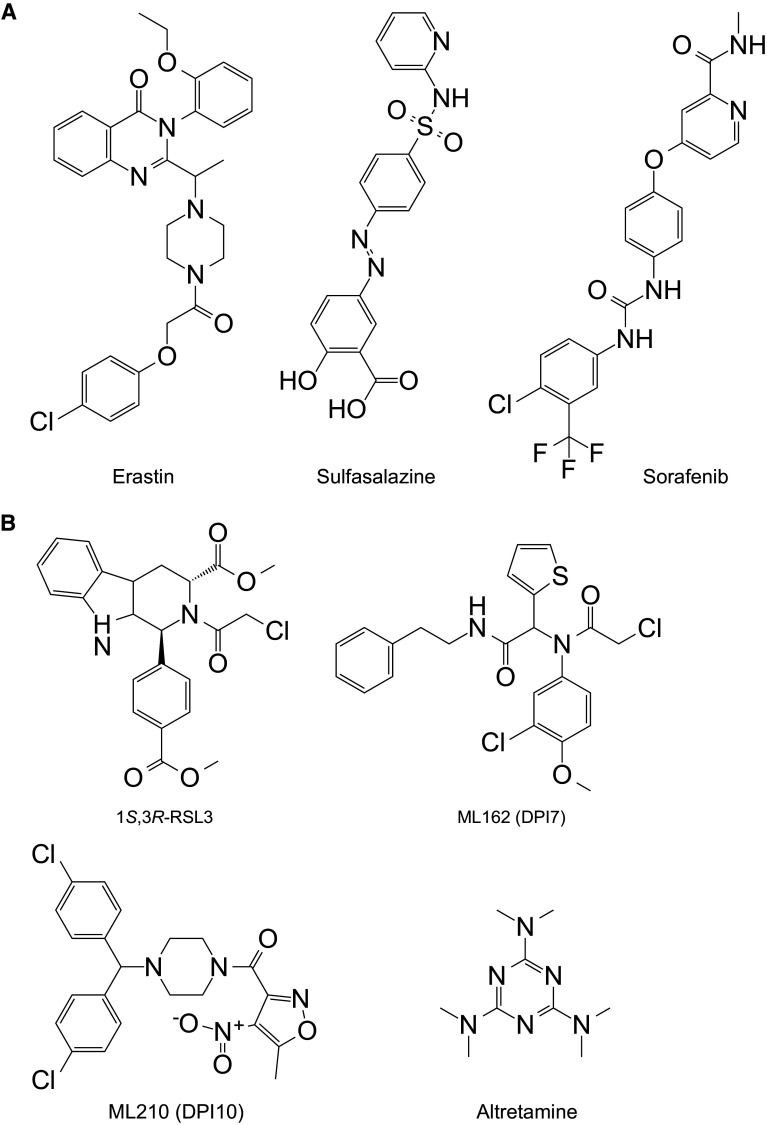
Structure of small molecule ferroptosis inducers. **a** molecules that inhibit the function of system $x_{\text{c}}^{ - }$. **b** Molecules that inhibit the function of glutathione peroxidase 4 (GPX4). ML162 is also known as DPI7

The RAS family of small GTPases (HRAS, NRAS and KRAS) is commonly mutated in cancer and several groups have searched for small molecules that are selectively lethal to cells expressing oncogenic mutant RAS proteins [12, 14–16]. In the 2000’s, the Stockwell laboratory isolated two novel oncogenic RAS Selective Lethal (RSL) small molecules named eradicator of Ras and ST (erastin) and Ras Selective Lethal 3 (RSL3) (Fig. 2a, b) [12, 17]. Both compounds were lethal at lower doses in engineered human tumor cells expressing oncogenic HRAS^V12^ than in isogenic cells expressing wild-type HRAS [12, 17]. The recognition of ferroptosis as a unique form of RCD emerged, unexpectedly, from characterizing the lethal mechanism of action of erastin and RSL3.

Erastin and RSL3 treatment do not trigger morphological changes or biochemical processes consistent with apoptosis, such as chromatin margination or cleavage of poly ADP-ribose polymerase (PARP) [12, 13, 17]. Moreover, erastin- and RSL3-induced cell death is not attenuated by caspase inhibition, by deletion of the intrinsic apoptotic effectors BCL-2 associated X protein (BAX) and BCL-2 antagonist/killer 1 (BAK), by a small molecule inhibitor of necroptosis (e.g. necrostatin-1) or by inhibition of autophagy (e.g. using chloroquine, 3-methyladenine) [5, 6, 12, 17, 18]. Neither mitochondrial ROS production nor the influx of Ca^2+^ is essential for ferroptosis [5]. However, erastin treatment results in a unique ‘dysmorphic’ mitochondrial phenotype observable by transmission electron microscopy [5]. Erastin-induced cell death also depends on a unique set of genes compared to cell death or cytostasis triggered by pro-apoptotic or pro-necrotic agents [5]. Crucially, erastin- and RSL3-induced cell death is effectively inhibited by the iron chelators DFO, 311, ciclopirox (CPX) and 2,2-bipyridyl (2,2-BP), as well as by the lipophilic antioxidants trolox (a soluble vitamin E analog), butylated hydroxyanisole, butylated hydroxytoluene and the novel synthetic antioxidant ferrostatin-1 (Fer-1) [5, 13, 17] (Fig. 1). These results indicate that iron-dependent L-ROS accumulation is essential for erastin- and RSL3-induced cell death. On this basis, the unique erastin- and RSL3-induced cell death phenotype was named ‘ferroptosis’ [5] (Table 1).

## Early studies informing our understanding of the ferroptotic mechanism

The role of oxidative stress in cell death has been studied for some time. Pioneering studies in the 1950’s by Harry Eagle and colleagues examined the amino acids, vitamins and other nutrients required to support the growth and proliferation of mammalian cells in culture [19]. Among those determined to be essential was cystine (Cys_2_), the oxidized form of the thiol-containing amino acid cysteine (Cys) [19]. Cells deprived of Cys_2_ fail to grow unless cultured at extremely high densities [19–21]. Following up on these observations, in 1977 Shiro Banni and colleagues found that depriving cultured human lung fibroblasts of Cys_2_ resulted in rapid depletion of the Cys-containing antioxidant tripeptide GSH (γ-l-glutamyl-l-cysteinylglycine), and subsequent cell death [22]. Cell death was prevented, without rescuing GSH levels, by growing cells in the presence of the lipophilic antioxidant α-tocopherol (vitamin E) [22]. These results implied that Cys_2_ import was needed to sustain GSH levels, and that cell death was triggered by a buildup of L-ROS. In subsequent years, studies of Cys_2_ deprivation-induced cell death in human embryonic fibroblasts, neuronal hybridoma cells and rat oligodendrocytes confirmed the importance of GSH depletion in cell death, and demonstrated that both lipophilic antioxidants and iron chelators could block this process from occurring [23–26]. Collectively, these reports established that continuous Cys_2_ uptake and GSH synthesis are required in many types of mammalian cells to prevent the accumulation of toxic L-ROS and help frame our understanding of how erastin and RSL3 trigger ferroptosis at the molecular level.

## Inhibition of system $x_{\text{c}}^{ - }$ triggers ferroptosis

Analysis of the erastin mechanism of action provided the first insights into proteins and pathways necessary to prevent the onset of ferroptosis. Early chemoproteomic studies using erastin analogs conjugated to a solid support matrix identified the mitochondrial voltage dependent anion channel 2 and 3 (VDAC2 and VDAC3) as direct erastin targets [13]. Experiments using purified human VDAC2 reconstituted into artificial liposomes confirm that erastin can bind this target and modulate transport flux [27]. However, it now appears that the ability of erastin to trigger ferroptosis is determined mainly by inhibition of a different target, the cystine/glutamate antiporter termed system $x_{\text{c}}^{ - }$ [5, 28] (Fig. 1).

System $x_{\text{c}}^{ - }$ is a heterodimeric cell surface amino acid antiporter composed of the twelve-pass transmembrane transporter protein SLC7A11 (xCT) linked by a disulfide bridge to the single-pass transmembrane regulatory protein SLC3A2 (4F2hc, CD98hc) [29]. System $x_{\text{c}}^{ - }$ imports extracellular Cys_2_ in exchange for intracellular Glu. Using ‘modulatory profiling’ (see [18]) it was found that cell death induced by erastin is similar in many respects to cell death induced by sulfasalazine (SAS) [5], a known system $x_{\text{c}}^{ - }$ inhibitor [30] (Fig. 2a) (Table 2). Notably, the lethal effects of both SAS and erastin are reversed by co-treatment with β-mercaptoethanol (β-ME) [5, 28], which bypasses the need for system $x_{\text{c}}^{ - }$, by forming mixed disulfides with Cys_2_ that can be imported into the cell by a different transporter [31]. Most convincingly, erastin and SAS block the uptake of radiolabelled Cys_2_ in cultured cancer cells [5, 28]. Thus, erastin appears to act as a direct inhibitor of system $x_{\text{c}}^{ - }$ function. This links the erastin mechanism of action to a process (Cys-dependent GSH synthesis) that normally opposes the accumulation of L-ROS. Indeed, erastin treatment leads to significant depletion of intracellular GSH, as detected using traditional biochemical methods and more advanced metabolomics analyses [6, 7, 28]. It is not known precisely how erastin or SAS inhibit SLC7A11-mediated Cys_2_ import. It was initially proposed that erastin bound to a related transport protein, SLC7A5, and inhibited SLC7A11 *in* *trans* [5]. However, more recent data argue against this possibility, and suggest that erastin most likely inhibits SLC7A11 directly [28]. More potent and drug-like analogs of erastin have been described and should facilitate future studies of the targets and effects of erastin in vitro and in vivo [6, 28, 32].

**Table 2:** Examples of small molecule-induced ferroptosis

Small molecule	Cell line	Target	Observation	References
Erastin, sulfasalazine	Engineered human tumor cells, HT-1080, Calu-1, A-673, Panc-1, other cancer cell lines, isolated mouse renal tubules	System $x_{\text{c}}^{ - }$	Death suppressed by iron chelators (DFO, CPX), lipophilic antioxidants (e.g. Fer-1, trolox, vitamin E), the protein synthesis inhibitor cycloheximide, the reducing agent beta-mercaptoethanol, the transaminase inhibitor amino-oxyacetate	Dixon et al. [5], Linkermann et al. [8], Dolma et al. [12], Yagoda et al. [13], Eling et al. [15], Yang and Stockwell [17]
Sorafenib	HT-1080, Huh7, ACHN	System $x_{\text{c}}^{ - }$	Death suppressed by DFO, Fer-1	Dixon et al. [28], Lachaier et al. [33], Louandre et al. [34]
1*S*,3*R*-RSL3, DPI19, DPI18, DPI17, DPI13, DPI12, DPI10 (ML210), DPI7 (ML162), altretamine	Engineered human tumor cells, HT-1080, Calu-1, others	GPX4	Death suppressed by iron chelators (311, DFO, CPX) and lipophilic antioxidants (butylated hydroxytoluene, trolox, vitamin E, Fer-1)	Yang et al. [6], Dixon et al. [5], Yang and Stockwell [17], Woo et al. [40]
Artesunate	Panc-1	Possibly lysosomal iron; unknown	Death suppressed by lipophilic antioxidant (Fer-1) and iron chelation (DFO)	Eling et al. [15]
Buthionine-(*S*,*R*)-sulfoximine	MEFs, HT-1080	GCLC	Death suppressed by lipophilic antioxidant (α-tocopherol, Fer-1) and iron chelation (DFO)	Friedmann Angeli et al. [9], Seiler et al. [39]

*MEFs* mouse embryonic fibroblasts, *BSO* buthionine-(*S*,*R*)-sulfoximine, *GCLC* glutamate-cysteine ligase, catalytic subunit, *DFO* deferoxamine

In addition to erastin and SAS, the FDA-approved multi-kinase inhibitor sorafenib (trade name: Nexavar) can block system $x_{\text{c}}^{ - }$ function, deplete GSH and trigger ferroptosis in cancer cell lines derived from liver, kidney, bone, lung and other tissues [28, 33, 34] (Table 2) (Fig. 2a). Related kinase inhibitors have no ability to block system $x_{\text{c}}^{ - }$ function or cause ferroptosis [28, 33], suggesting that the effects of sorafenib could be due either to modulation of a very specific kinase (that in turn modulates system $x_{\text{c}}^{ - }$ activity) or to a direct effect on system $x_{\text{c}}^{ - }$. This function may explain the ability of sorafenib to trigger caspase-independent cell death in certain cell types and enhance ROS accumulation in sorafenib-treated cancer patients [35, 36]. Indeed, it is intriguing to speculate that the clinical benefit of sorafenib observed in patients may be due, at least in part, to the activation of ferroptosis in vivo [28]. However, the effects of this compound are clearly pleiotropic: in some cell lines sorafenib triggers apoptosis [37], and even in cell lines where ferroptosis is observed at low doses of sorafenib, apoptosis or some other form of cell death is observed at higher doses [28]. Further study is required to disentangle the various effects of sorafenib on the cell and determine whether the effects of this compound in patients are attributable to ferroptosis.

## The role of GPX4 in preventing ferroptosis

Elucidation of the RSL3 mechanism of action provided the next major insight into the regulation of ferroptosis. Of the four possible RSL3 diastereomers, only one—1*S*,3*R*-RSL3—is capable of inducing ferroptosis [6] (Fig. 2B). Chemoproteomic studies using the active isomer of RSL3 as an affinity reagent identified the selenoprotein glutathione peroxidase 4 (GPX4, PHGPx) as a candidate target of this compound [6] (Fig. 1). GPX4 is a GSH-dependent enzyme that reduces lipid hydroperoxides (L-OOH) to lipid alcohols (L-OH). GPX4, therefore, normally limits the iron-dependent formation of highly reactive lipid alkoxy radicals (L-O^·^) from L-OOH [38, 39]. Cells appear to be continually exposed to the threat of L-ROS-mediated destruction, as inhibition of GPX4 activity leads to the rapid accumulation of L-ROS and cell death in cell culture, and deletion of *Gpx4* in mice is embryonic lethal [6, 39]. Consistent with RSL3-mediated inactivation of GPX4 being essential to induce ferroptosis, overexpression of *GPX4* blocks RSL3-induced cell death while short hairpin RNA (shRNA)-mediated knockdown of *GPX4* in human oncogenic HRAS cells is sufficient to induce ferroptotic cell death [6]. Deletion of *Gpx4* in mouse cells also results in cell death that can be suppressed by lipophilic antioxidants (e.g. Fer-1) and iron chelators, further confirming that GPX4 activity is essential to prevent ferroptosis [9, 39].

In addition to RSL3, nine other synthetic small molecules, including ML162 (also known as DPI7), ML210 (also known as DPI10) and, most unexpectedly, the FDA-approved anticancer agent altretamine, can inhibit GPX4 activity [6, 16, 40] (Table 2; Fig. 2b). Like RSL3, these compounds inhibit GPX4 enzymatic activity without depleting the cell of glutathione. Thus, RSL3 and functionally related compounds are classified as “class 2” ferroptosis-inducing compounds (FINs), to distinguish them from erastin and other system $x_{\text{c}}^{ - }$ inhibitors that that most likely block GPX4 function indirectly by preventing GSH synthesis (“class 1” FINs) [6, 40]. Mechanistically, how RSL3 and other class 2 FINs bind GPX4 to inhibit its activity is not known.

## Ferroptosis can be induced by glutathione depletion

Ferroptosis can be induced by depriving cells of the essential GSH precursor, Cys, or by blocking the function of the GSH-dependent enzyme GPX4 (see above). Thus, direct inhibition of GSH synthesis would be predicted to trigger ferroptosis. Consistent with this prediction, inhibition of glutamate-cysteine ligase (GCL, formerly known as γ-glutamylcysteine synthetase), the rate-limiting first enzyme in the two-step synthesis of GSH, using buthionine-(*S*,*R*)-sulfoximine (BSO [41]), can induce cell death that is suppressed by α-tocopherol and DFO, but not by the caspase inhibitor zVAD-fmk or the necroptosis inhibitor Nec-1 [9, 39]. This suggests that inhibition of GSH synthesis is sufficient to trigger ferroptosis, at least in some cells (Fig. 1). Curiously, however, in many ferroptosis-sensitive cells BSO is a far less potent inducer of ferroptosis than inhibition of system $x_{\text{c}}^{ - }$ or GPX4 (J. Cao & S. Dixon, *unpublished*). One possibility is that direct GCL inhibition leads to upregulation of an alternative antioxidant pathway that can maintain cell survival in the absence of GSH. For example, high levels of SLC7A11-mediated Cys_2_ import, in conjunction with the GSH-independent thioredoxin (Txn) system, can substitute for the essential function of the GSH-GPX4 lipid peroxide metabolic pathway in some cells both in vitro and in vivo [42–44]. Mechanistically, this might involve the transfer of reducing equivalents from Cys_2_ to Txn (via thioredoxin reductase) in sufficient quantities to maintain the endogenous lipid antioxidant α-tocopherol in a reduced state, and thereby, prevent l-ROS accumulation [45]. Regarding the connection between GSH and cell death, a major issue remaining to be resolved concerns the type of cell death induced by GSH depletion. As described above, in some cells GSH depletion can trigger ferroptosis. However, GSH depletion has also been associated with the induction of apoptosis and sensitization to apoptosis-inducing agents (e.g., SMAC mimetics) [46–48]. While more work is required to reconcile the latest findings concerning the role of GSH in ferroptosis with the literature linking GSH depletion and apoptosis, a speculative model is that the depletion of cytosolic GSH promotes ferroptosis, while the depletion of the separate mitochondrial GSH pool promotes, or is more closely associated with, apoptosis [9, 49].

## The causes of ferroptotic cell death downstream of GSH depletion and GPX4 inactivation

In response to system $x_{\text{c}}^{ - }$ inhibition or GPX4 inactivation, ferroptotic cell death involves the iron-dependent accumulation of L-ROS and the depletion of polyunsaturated fatty acids (PUFAs) [5–7, 9]. L-ROS are typically formed from the PUFA chains of membrane lipids. PUFAs are susceptible to both enzymatic (e.g., lipoxygenase-catalyzed) and non-enzymatic (e.g., ROS-catalyzed) oxidation, leading to the formation of lipid hydroperoxides (L-OOH) [50]. In the presence of iron, L-OOH can form toxic lipid radicals such as the alkoxy radical L-O^·^. These lipid radicals can abstract protons from adjacent PUFAs, initiating a new round of lipid oxidation and further propagation of oxidative damage from one lipid to another [50]. PUFA oxidation and free-radical-mediated damage can ultimately result in PUFA fragmentation into a variety of products [50]. In erastin-treated cancer cells and *Gpx4* null mouse cells, L-ROS accumulation, PUFA depletion and cell death are all prevented by treatment with small molecule antioxidants such as Fer-1, suggesting that lipid ROS-mediated damage is essential for ferroptosis [5, 7, 9]. The recently described ferroptosis inhibitor, liproxstatin-1, may also function as a lipophilic antioxidant, although the mechanism of action of this inhibitor has yet to be reported [9].

In cells undergoing ferroptosis the PUFA arachidonic acid (AA) is significantly depleted and AA-derived lipid fragments are detected in the supernatants of *Gpx4* ^−/−^ mouse embryonic fibroblasts (MEFs) [7, 9]. Consistent with a key role for AA in ferroptosis, the deletion of two genes, acyl-CoA synthetase long-chain family member 4 (*ACSL4*) and lysophosphatidylcholine acyltransferase 3 (*LPCAT3*), prevents ferroptosis induced by the GPX4 inhibitors RSL3 and ML162 [51]. *ACSL4* and *LPCAT3* encode enzymes involved in the insertion of AA into membrane phospholipids [52, 53]. This suggests that the execution of ferroptosis can only proceed, following direct or indirect (i.e., GSH depletion-induced) inactivation of GPX4, when highly oxidizable PUFAs such as AA are present in the membrane.

The molecular events that occur downstream of PUFA oxidative fragmentation to cause irreversible cell death are unclear. PUFA fragmentation and membrane lipid damage may be sufficient to irreversibly permeabilize the plasma membrane. Alternatively or in parallel, reactive lipid intermediates generated following PUFA oxidation could promote cell death by covalently modifying and inactivating essential intracellular proteins. In support of this possibility, cell lines selected for resistance to ferroptosis overexpress three aldo–keto reductase family 1, member C (*AKR1C*) family members [28]. These proteins can detoxify the toxic reactive lipid intermediate 4-hydroxynonenal (4-HNE), which can be formed downstream of oxidative PUFA fragmentation [54, 55]. A plausible model is that the activity of AKR1C family members can restrain ferroptosis by preventing the accumulation of 4-HNE to toxic levels, but functional studies are required to investigate this hypothesis in detail.

## The role of iron in ferroptosis

Iron is essential for the execution of ferroptosis. Both membrane permeable (e.g. CPX, 311 and 2,2-BP) and membrane impermeable (e.g. DFO) iron chelators prevent cells from undergoing ferroptosis, whether induced by erastin, RSL3 or a physiological stimulus such as high concentrations of extracellular Glu [5, 6, 17]. Likewise, ferroptosis induced by erastin or Cys_2_ deprivation is prevented by genetic silencing of *TFRC*, which encodes the transferrin receptor required for the uptake of transferrin-iron complexes into the cell [10, 17]. Conversely, supplementing the growth medium with iron-bound transferrin or a bioavailable form of iron (e.g., ferric ammonium citrate), but not other divalent metals, accelerates erastin-induced ferroptosis [5, 10]. These results firmly establish the need for iron in ferroptosis.

How iron promotes ferroptosis inside the cell remains unclear. Although a redox-independent role for iron cannot formally be ruled out, the most obvious explanation for the ability of iron chelators to block ferroptosis is that they prevent iron from donating electrons to oxygen to form ROS [56]. The properties of different iron chelator classes provide some insights. Lipophilic iron chelators can cross the plasma membrane and chelate the intracellular free, ‘redox active’ iron pool [57]. This may block death by preventing this iron pool from catalyzing the formation of soluble or lipid radicals that can initiate or propagate oxidative PUFA fragmentation, respectively [50]. Alternatively or in parallel, lipophilic iron chelators may directly inactivate iron-containing enzymes that promote membrane lipid oxidation. In this connection, the lipoxygenase (LOX) family of enzymes are interesting candidates to mediate iron-dependent L-ROS formation. The iron-dependent LOX enzymes catalyze site-specific oxidation of PUFAs such as AA, and are directly inactivated by lipophilic iron chelators [58–60]. Small molecule LOX inhibitors block cell death due to depletion of GSH or deletion of *Gpx4* [39, 61, 62]; however, protection from *Gpx4* deletion is not observed when different Lox enzymes are inactivated using genetic reagents individually or in combination [9, 39]. Other iron-dependent enzymes, including the iron and 2-oxoglutarate-dependent dioxygenase prolyl 4-hydroxylase isoform 1 (PHD1) may also be a relevant target of iron chelator action in the prevention of GSH depletion-induced cell death [63]. However, a molecular mechanism linking PHD1 function to the production of L-ROS is less obvious than for LOX enzymes which are known to oxidize PUFAs directly.

Unlike lipophilic iron chelators, DFO is a membrane impermeable iron chelator that accumulates in the lysosome through endocytosis [59]. This suggests that DFO likely prevents ferroptosis by chelating lysosomal iron. However, unlike lethal treatments such as H_2_O_2_, that cause the destruction of the lysosome [64], there is no evidence that ferroptosis-inducing compounds such as erastin trigger lysosomal bursting [7]. Thus, a reasonable model is that DFO, acting within the lysosome, intercepts iron that is ultimately destined for another location in the cell more directly responsible for promoting L-ROS formation (e.g., the ‘redox active’ pool and/or a specific iron-dependent enzyme).

Iron chelation not only prevents ferroptosis, but also cell death induced by H_2_O_2_ and the synthetic compound artesunate [5]. However, cell death in response to these lethal triggers is typically not blocked by ferroptosis-specific antioxidants such as Fer-1 [5], suggesting that in most cells these agents do not induce ferroptosis *per se* (but see [65]). Therefore, the combined use of both iron chelators and lipophilic antioxidants is required to definitively assess the role of ferroptosis in a particular lethal phenotype.

## Modulators of ferroptosis

Hypothesis-driven investigations and unbiased loss-of-function genetic screens have identified genes that are essential for, or that modulate the sensitivity to, ferroptosis (Table 3). Ferroptosis was originally characterized through the study of compounds (i.e., erastin, RSL3) that are selectively more lethal to oncogenic RAS mutant cancer cells. Additional RSL compounds have been identified on the basis of this cellular phenotype, and subsequently confirmed to trigger ferroptosis (e.g. [6, 16]). These results suggest a relationship between ferroptosis and oncogenic RAS activity, at least in certain cells. In KRAS-mutant Calu-1 lung cancer cells shRNA-mediated silencing of *KRAS* reduces sensitivity to erastin [13]. Silencing of oncogenic mutant BRAF in A-673 cells also reduces sensitivity to erastin [13], suggesting that the activity of the broader RAS-RAF-MEK pathway could determine ferroptosis sensitivity in individual cell lines. Constitutive RAS pathway activity can promote the expression of TFRC and suppress the expression of iron storage proteins in engineered tumor cell lines, providing one explanation for how oncogenic RAS activity could promote sensitivity to ferroptosis [17]. However, this model remains to be tested in additional cell types.

**Table 3:** Genes and proteins identified as mediators or modulators of ferroptosis

Gene	Identification method	Gene product	Gene product function	References
*TFRC*	Candidate gene, RNAi	Transferrin receptor	Import of transferrin-iron complexes	Yang and Stockwell [17], Gao et al. [10]
*ACSF2*	shRNA screen	Acyl-CoA synthetase family member 2	Fatty acid metabolism	Dixon et al. [5]
*EMC2/TTC35*	shRNA screen	ER membrane protein complex subunit 2	Unknown. Possible role in protein folding in the endoplasmic reticulum	Dixon et al. [5]
*RPL8*	shRNA screen	Ribosomal protein L8	Core component of the ribosomal large subunit involved in protein synthesis.	Dixon et al. [5]
*IREB2*	shRNA screen	Iron-responsive element binding protein 2	Master regulator of iron homeostasis	Dixon et al. [5]
*SLC7A11*	Candidate gene approach	Solute carrier family 7 (anionic amino acid transporter light chain, xc- system), member 11	Cystine/glutamate antiporter.	Dixon et al. [5]
*CS*	shRNA screen	Citrate synthase	Lipid metabolism	Dixon et al. [5]
*ATP5G3*	shRNA screen	ATP synthase, H + transporting, mitochondrial F_o_ complex, subunit C3 (subunit 9)	Complex V of mitochondrial F_o_F_1_ ATPase; ATP synthesis	Dixon et al. [5]
*GPX4*	Candidate gene approach	Glutathione peroxidase 4	Lipid repair	Yang et al. [6]
*GCLC*	Candidate gene approach	Glutamate-cysteine ligase, catalytic subunit	Glutathione synthesis	Yang et al. [6]
*ACSL4*	Human haploid cell genetic screen	Acyl-CoA synthetase long-chain family member 4	Lipid metabolism	Dixon et al. [51]
*LPCAT3*	Human haploid cell genetic screen	Lysophosphatidylcholine acyltransferase 3	Lipid metabolism	Dixon et al. [51]
*CARS, EPRS, HARS*	Genome-wide siRNA screen	Cysteinyl-tRNA synthetase	Protein translation	Hayano et al. [67]
*SLC1A5*	Candidate gene approach	Solute carrier family 1 (neutral amino acid transporter), member 5	Glutamine transport	Gao et al. [10]
*GLS2*	Candidate gene approach	Glutaminase 2 (liver, mitochondrial)	Glutaminolysis	Gao et al. [10]
*GOT1*	Candidate gene approach	Glutamic-oxaloacetic transaminase 1, soluble	Glutaminolysis	Gao et al. [10]
*HSPB1*	Candidate gene approach	Heat shock 27 kDa protein 1	Protein folding; iron metabolism	Sun et al. [87]
*TP53*	Candidate gene approach	Tumor protein p53	Tumor suppressor, metabolic regulator	Jiang et al. [11]

The link between RAS pathway activity and ferroptosis is complicated by two observations. First, when comparing profiles of erastin sensitivity across a panel of 117 cancer cell lines, RAS-mutant cancer cell lines are on average no more sensitive to ferroptosis-inducing compounds than cancer cells expressing wild-type RAS [6]. In fact, for unknown reasons, diffuse large B cell lymphoma (DLBCL) and renal cell carcinoma cancer cell lines, which do not typically contain RAS pathway mutations, emerge as the most sensitive types of cancer cells [6]. Second, RMS13 rhabdomyosarcoma cells overexpressing oncogenic *HRAS*, *KRAS* or *NRAS* are resistant to erastin and RSL3 [66]. A reasonable explanation for this confusing picture is that the effects of RAF-MEK-ERK pathway activity on ferroptosis differ depending on cell lineage or mutant RAS protein expression levels. The discovery of biomarkers that more universally predict sensitivity and resistance to ferroptosis would help guide the development of these agents for cancer treatment.

In addition to RAS pathway components, additional genes have been found to modulate ferroptosis (see Table 3). These genes can be linked to processes known to be essential for ferroptosis, including iron metabolism (*TFRC*, *IREB2*, *HSPB1*), protein synthesis (*RPL8*) and lipid metabolism (*ACSF2*, *ACSL4*, *LPCAT3*, and possibly *CS*). Silencing of *CARS*, and certain other tRNA synthetases (*HARS*, *EPRS*), appears to promote cell survival indirectly, by enhancing the synthesis of Cys from methionine, via the transsulfuration pathway, allowing the cell to maintain GSH synthesis when system $x_{\text{c}}^{ - }$ is blocked by erastin [67]. *SLC1A5*, *GLS2* and *GOT1* are required for glutamine uptake and metabolism to Glu and, ultimately, α-ketoglutarate [10]. The role of Gln metabolism in ferroptosis is not clear, although this pathway may contribute to the formation of oxidizable membrane lipids by feeding precursors (i.e., citrate) towards fatty acid or lipid synthesis [5]. The role of other genes (*ATP5G3*, *EMC2*/*TTC35*) in ferroptosis has yet to be studied in detail.

Another recently described modulator of ferroptosis is p53, encoded by *TP53*. Using a p53-inducible cell line, p53 upregulation was shown to repress expression of the system $x_{\text{c}}^{ - }$ transporter subunit *SLC7A11* and sensitize cells to ferroptosis [11]. Chromatin immunoprecipitation and electrophoretic mobility shift analysis experiments suggest that p53 binds to the *SLC7A11* locus at a specific p53 response element within the 5’ untranslated region. At this site, p53 presumably recruits chromatin-modifying enzymes that repress *SLC7A11* transcription. These results are of great interest since the mechanism of p53-mediated tumor suppression remains highly controversial. While early literature suggested that p53-dependent tumor suppression involved the induction of cell cycle arrest, senescence or apoptosis, the recent analysis of several transactivation-defective p53 mutants has called this model into question and it now appears that alterations in intracellular metabolism may account for the ability of p53 to suppress tumor formation [68–70]. p53-dependent effects on *SLC7A11*-mediated Cys_2_ uptake fit within this emerging picture of p53-dependent metabolic modulation, although it should be noted that *SLC7A11* was not identified in other comprehensive studies of genomic p53 binding sites and direct p53 transcriptional targets [71, 72]. Moreover, p53 is known to induce the expression of a number of antioxidant genes that would be predicted to suppress ROS accumulation, and therefore counteract ferroptosis [70]. Resolving the role of p53 in ferroptosis promises to be an active area of investigation.

## The role of ferroptosis in pathological cell death

A role for ferroptosis has been found in a growing number of pathological cell death scenarios (Table 4). These studies have been enabled by the discovery of novel small molecules, including Fer-1 [5], improved Fer-1 analogs [7, 8] and liproxstatin-1 [9], that potently and specifically block ferroptosis. In a rat hippocampal slice culture model, exposure to high concentrations of Glu triggers substantial cell death that can be significantly attenuated by both Fer-1 and the iron chelator CPX [5]. Complete protection from cell death is observed with Fer-1 and several Fer-1 analogs in rat oligodendrocytes deprived of Cys_2_, a model of the pathological process leading to periventricular leukomalacia, while more modest protection is observed in a model of Huntington’s disease and in a model of iron-induced kidney tubule damage [7]. Fer-1 and improved analogs also protect isolated renal tubules from erastin-induced cell death, reduce kidney injury following acute oxalate-induced damage, and protect from acute renal failure and organ damage in a model of severe kidney ischemia/reperfusion injury [8]. In a similar vein, the novel ferroptosis inhibitor liproxstatin-1 attenuates cell death in, and extends survival of, mice in which *Gpx4* is selectively deleted from the kidney [9]. Finally, both DFO and the glutaminolysis inhibitor Compound 968 prevent cell death in an ex vivo model of ischemia/reperfusion injury in the mouse heart [10]. While these results are suggestive of ferroptosis in this pathological scenario, further work using ferroptosis-specific inhibitors such as Fer-1 will be helpful to confirm this point.

**Table 4:** Known and suspected physiological or pathological ferroptosis-inducing conditions

Treatment	System	Observation	References
Glutamate	Rat postnatal hippocampal slice culture	Death suppressed by Fer-1, CPX	Dixon et al. [5]
Cystine deprivation	Rat postnatal pre-oligodendrocyte cultures	Death suppressed by Fer-1	Skouta et al. [7]
Huntington gene fragment overexpression	Transfected postnatal corticostrial rat brain slice	Death suppressed by Fer-1	Skouta et al. [7]
Iron overload	Mouse kidney proximal tubules	Death suppressed by Fer-1	Skouta et al. [7]
Acetaminophen	Mouse hepatocytes	Death suppressed by Fer-1	Lőrincz et al. [88]
*Gpx4* deletion	MEFs, mouse kidney cells, mouse T cells	Rapid death, suppressed by vitamin E, Fer-1	Friedmann Angeli et al. [9], Matsushita et al. [89], Seiler et al. [39]
p53 upregulation	MEFs	p53 upregulation leads to sensitization to ferroptosis	Jiang et al. [11]
Ischemia/reperfusion	Mouse kidney (in vivo), mouse heart (ex vivo)	Death suppressed by Fer-1 analogs, iron chelation	Gao et al. [10], Linkermann et al. [8]
Amino acid deprivation in presence of serum and glucose	MEFs	Death suppressed by Fer-1	Gao et al. [10]

*MEFs* mouse embryonic fibroblasts, * β-ME* beta-mercaptoethanol

These studies suggest a number of tissues and scenarios where the induction of ferroptosis may contribute to pathological cell death. One concern with the ex vivo model studies is that ambient levels of oxygen (O_2_, i.e., 21 %) artificially enhances any oxidative cell death process under consideration. While this cannot be completely ruled out, studies of mouse and human cells show that both erastin treatment and *Gpx4* inactivation trigger ferroptosis with similar inhibition profiles and cell death phenotypes at both ambient (i.e., 21 %) and physiological (<5 %) levels of O_2_, suggesting that ferroptotic mechanisms remain active in low oxygen conditions [28, 39]. A second concern associated with these studies is that it is impossible to know, with certainty, that Fer-1 and other inhibitors are blocking ferroptosis and not another form of cell death. This is because we currently lack suitable molecular markers of ferroptosis that would identify cells undergoing this process, prior to death. While the mRNA expression levels of two genes prostaglandin E synthase 2 (*PTGES2*) and ChaC glutathione-specific gamma-glutamylcyclotransferase 1 (*CHAC1*) are significantly elevated in cells undergoing ferroptosis [6, 28], these are not suitable for use in live cells or intact tissues. Further work is needed to identify additional ferroptotic markers that could be used for future in vivo studies.

## Ferroptosis and oxidative glutamate toxicity

Ferroptosis appears similar in several respects to oxidative glutamate toxicity (OGT), a phenotype observed in certain neuronal cell lines treated with high concentration of Glu to inactivate system $x_{\text{c}}^{ - }$ and deprive the cell of Cys_2_ (e.g. [25, 73–75]). Both erastin-induced ferroptosis and OGT involve GSH depletion, L-ROS accumulation and cell death that can be blocked by lipophilic antioxidants including Fer-1 [5, 25, 62, 73, 76, 77]. However, as discussed previously (see [5, 56]), a number of key differences between ferroptosis and OGT are apparent, downstream of L-ROS accumulation. For example, in OGT, but not ferroptosis, extracellular Ca^2+^ influx, BH3 interacting domain death agonist (Bid)-mediated mitochondrial damage and nuclear translocation of apoptosis inducing factor 1 (AIF1) are essential for death [62, 73, 78]. Thus, while ferroptosis and OGT can share a common mechanism of initiation (i.e. Cys_2_ deprivation), the terminal phases of death execution appear to be more complex and elaborate in cells undergoing OGT. Of note, AIF1 is reportedly essential for cell death in MEFs lacking *Gpx4* [39], although this result remains to be confirmed in other cells. Whether ferroptosis is an abbreviated form of OGT specific to cancer cells, or whether OGT is a more elaborate form of ferroptosis specific to neuronal cells, remains to be resolved.

## Conclusions and perspectives

**Fig. 3:**
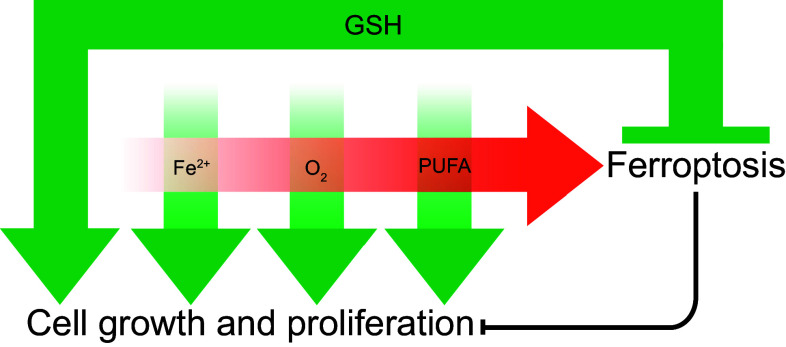
The relationship between iron, diatomic oxygen (O_2_), PUFAs and glutathione (GSH). Iron (Fe^2+^), O_2_ and PUFAs are each, individually, required for cell growth and survival (*green arrows*). GSH is also required for cell growth and proliferation, as well as to prevent the combination of Fe^2+^, O_2_ and PUFAs from triggering ferroptosis

Ferroptosis lies at the nexus of essential biological processes involving O_2_, iron and PUFAs. O_2_, iron and PUFAs are, individually, essential for cell growth and proliferation. However, the interaction between O_2_, iron and PUFAs can lead to the accumulation of toxic levels of L-ROS. Thus, ferroptosis results from an imbalance between O_2_-dependent, iron-catalyzed, L-ROS production and GSH-dependent GPX4 activity (Fig. 3). Compared to other forms of RCD, the ‘logic’ of the ferroptosis pathway is unique. Apoptosis, for example, is triggered by diverse lethal stimuli (e.g. DNA damage, protein misfolding, etc.) leading to activation of a latent, pro-death enzymatic program and the ordered disassembly of the cell. Ferroptosis, by contrast, results from the inactivation of an essential metabolic process, leading to an iron-catalyzed, L-ROS-mediated cellular collapse. It has been noted by Green and Victor that ferroptosis is therefore best described as a form of cellular ‘sabotage’, wherein the normal metabolic functions of the cell contribute to cell death [79]. This distinguishes ferroptosis from apoptosis and other forms of RCD that are best described as ‘cell suicide’ [79]. Whether the inactivation of any other essential metabolic processes can trigger ferroptosis, or perhaps other novel cell sabotage programs, is unknown.

The distinction between ferroptosis as a form of cell sabotage and apoptosis as a form of cell suicide is intriguing, but begs the question of why cell sabotage might exist in the first place. Why does GSH depletion and GPX4 inactivation not simply trigger apoptosis? Is there something unique about GSH depletion and/or GPX4 inactivation that would make an alternative form of death like ferroptosis inevitable? One possibility is that GSH is required for the execution of apoptosis and that GSH depletion therefore inhibits the apoptotic program. While certain cancer cells treated with BSO (to deplete GSH) are unable to activate apoptosis in response to lethal doses of various alkylating agents, GSH is apparently not required for apoptotic cell death in response to most lethal triggers [80, 81]. Thus, it seems that cells depleted of GSH are fully capable of undergoing apoptosis, and yet still typically adopt a ferroptotic fate.

An alternative model is that GSH depletion is ‘insulated’ from the apoptotic pathway to enable it to produce a specific, adaptive cellular phenotype. In cells undergoing ferroptosis caspases are not activated [13] and dying cells are therefore likely to release a number of immune modulators [8]. This may be beneficial if the goal is to trigger an immune response. Interestingly, the system $x_{\text{c}}^{ - }$ transporter SLC7A11 is required for entry of Kaposi’s sarcoma-associated herpesvirus into certain cells [82], a process likely to disrupt normal glutathione homeostasis. Likewise infection of immune cells with the human immunodeficiency virus type-1 (HIV-1) is well know to cause dysregulation of glutathione homeostasis [83, 84], possibly enhancing sensitivity to ferroptosis. Thus, ferroptosis may be a fate preferentially adopted by certain immune cells, in response to infection, to ensure the release of appropriate immunostimulatory signals. More broadly, low levels of intracellular Cys and GSH may be sensed, like ATP and acetyl-CoA, as indicators of poor cell health and constitute a novel ‘metabolic checkpoint’ [85]. Cells with low levels of antioxidant defenses accumulate DNA damage more readily [86] and in multicellular organisms it could, therefore, be adaptive to have a specific route available for these cells to be eliminated. Future studies of ferroptosis promise to illuminate these and other questions surrounding this intriguing cell death process.
